# Supplementary figures and images for: Identification of the shared gene signatures and pathways between sarcopenia and type 2 diabetes mellitus
Source: PLoS One. 2022 Mar 10;17(3):e0265221. doi: 10.1371/journal.pone.0265221 (PMC8912249; doi:10.1371/journal.pone.0265221)

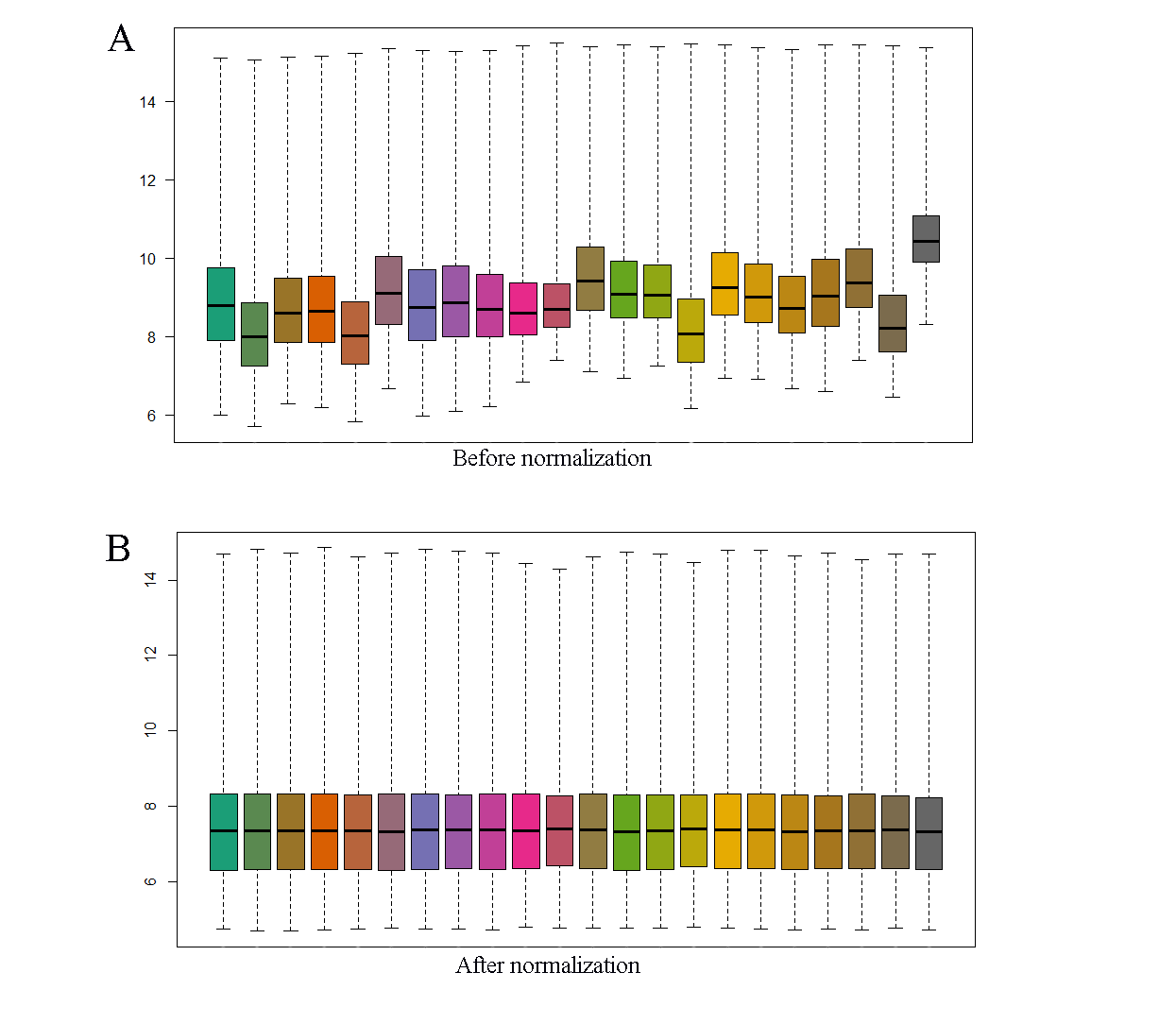

Supplement: S1 Fig — (TIF) [file pone.0265221.s001.tif]

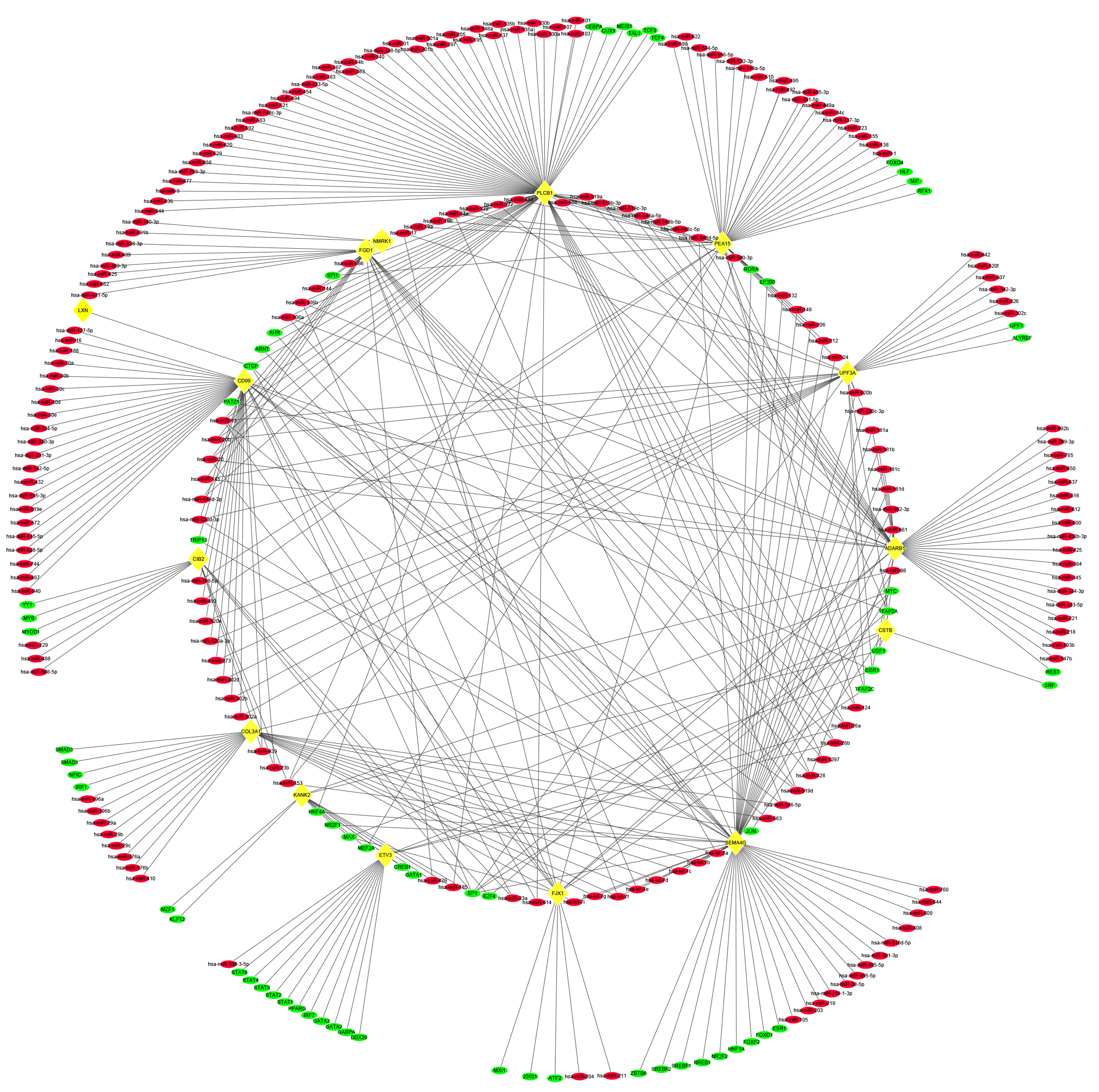

Supplement: S2 Fig — The nodes with yellow diamonds are common DEGs, red round nodes represent miRNA and green round nodes indicate TF. (TIF) [file pone.0265221.s002.tif]
