# Supplementary material for: Identification of the shared gene signatures and pathways between sarcopenia and type 2 diabetes mellitus
Source: PLoS One. 2022 Mar 10;17(3):e0265221. doi: 10.1371/journal.pone.0265221 (PMC8912249; doi:10.1371/journal.pone.0265221)
Supplement: Attachment — Submitted filename: Response to Reviewers.docx [file pone.0265221.s011.docx]

Dear Editor and Reviewers:

Thank you for taking time out of your busy schedule to review the manuscript. Now we have carefully corrected and replied the manuscript for this revision.

**Editor**

**Comment:** Please ensure that your manuscript meets PLOS ONE's style requirements, including those for file naming.

**Respond:** Corrections have been made.

**Comment:** Thank you for stating the following financial disclosure: “The National Natural Science Foundation of China (81901429).” Please state what role the funders took in the study. If the funders had no role, please state: "The funders had no role in study design, data collection and analysis, decision to publish, or preparation of the manuscript.” If this statement is not correct you must amend it as needed. Please include this amended Role of Funder statement in your cover letter; we will change the online submission form on your behalf.

**Respond:** Corrections have been made in the cover letter.

**Comment:** In your Data Availability statement, you have not specified where the minimal data set underlying the results described in your manuscript can be found. PLOS defines a study's minimal data set as the underlying data used to reach the conclusions drawn in the manuscript and any additional data required to replicate the reported study findings in their entirety. We will update your Data Availability statement to reflect the information you provide in your cover letter.

**Respond:** Corrections have been made in the cover letter.

**Comment:** We note that Figure 1 in your submission contain copyrighted images.

**Respond:** We have already changed Figure 1 to avoid the problem.

**Comment:** Please review your reference list to ensure that it is complete and correct. If you have cited papers that have been retracted, please include the rationale for doing so in the manuscript text, or remove these references and replace them with relevant current references. Any changes to the reference list should be mentioned in the rebuttal letter that accompanies your revised manuscript.

**Respond:** Thanks, we have double checked the reference list. Reference [30] and [31] has been placed after original reference [43] in revised manuscript.

**Reviewer 1**

**Comment:** First, the name of those miRNA which are regulating the selected hub genes are missing, network is so condensed that it is very difficult to figure out the name of those regulatory miRNA. It would be better if name of miRNA regulating these hub genes will be incorporated in result as well as discussion part.
Second, It would be much better if a subnetwork will be extracted from the main network of TF-miRNA-mRNA which specifically include only hub genes and its associated miRNA and TF. That would increase the clarity of network due to reduced nodes number and also will be more informative. Main network can be considered as supplementary data.

**Respond:** The number of miRNA which regulated the selected hub genes was large, so we have displayed them in S10 table. Since the network of main TF-miRNA coregulatory network is so condensed, we have exhibited it as S9 Fig and extracted a subnetwork involving only hub genes and its associated miRNA and TF as Fig 7.

**Comment:** Third, Why the author has decided to go with only one dataset in each case?

**Respond:** Of course, combining information from multiple existing studies can increase the reliability and generalizability of results. However, concerns also exist at the same time. Heterogeneities caused by demographic, clinical and technical variables often exist across studies from different platform. Failure to consider these variables in the statistical models and meta-analysis can result in reduced statistical power or false positives. In this study, we chose only GSE1428 for sarcopenia and GSE166467 for T2DM to get preliminary result. We should also notice the shortcoming of the study. Firstly, analytical methods may limit the predictive capability of the result and new proof from future studies may renew current results. Secondly, our analyses are based on public datasets, and further researches about the detailed molecular mechanism are necessary.

doi: [10.1371/journal.pmed.0050184](https://dx.doi.org/10.1371%2Fjournal.pmed.0050184)

doi: [10.1093/nar/gkr1265](https://dx.doi.org/10.1093%2Fnar%2Fgkr1265)

**Reviewer 2**

**Comment:** Some terms and concepts have been explained in the materials and methods section. It will be better if they are explained either in the introduction or the results section.

**Respond:** Corrections have been made. TFs and miRNA are explained in the discussion part in revised manuscript.

**Comment:** Lines 139, ‘and its inhibiting translation’, please rephrase. It is confusing.
**Respond:** Corrections have been made.

**Comment:** Line 153 should be ‘were obtained’.

**Respond:** Corrections have been made.

**Comment:** Line 218, Table 1, please use borders for all the cells. The information currently looks haphazard.

**Respond:** Corrections have been made.

**Comment:** Line 332, it is confusing. Please rephrase or clarify.

**Respond:** Corrections have been made in revised manuscript.

**Comment:** Line 354, should be ‘Acknowledgements’.

**Respond:** In standard American English, “acknowledgments” is spelled without the additional “e.” In British English, and most places outside of North America, “acknowledgements” is spelled with the extra “e.” After we searched some papers published on PLOS ONE recently, we found that ‘Acknowledgments’ without “e” was mainly used.

doi: 10.1371/journal.pone.0263301

[doi: 10.1371/journal.pone.0263344](https://doi.org/10.1371/journal.pone.0263344)

doi: [10.1371/journal.pone.0263307](https://dx.doi.org/10.1371%2Fjournal.pone.0263307)

Please do not hesitate to contact us if there are any question. Thanks again to the reviewers and editors for your hard work! Best wishes to you!

Yi Song

Department of Geriatrics, Union Hospital, Tongji Medical College, Huazhong University of Science and Technology, 1277 Jiefang Ave, Wuhan 430022, China.

Tel: +86-15629054511

Email: yisong[@hust.edu.cn](mailto:2017xh0147@hust.edu.cn)
